# Safety Assessment of Repeated Oral Administration of Tolfenamic Acid in Japanese Quails: Haematological, Biochemical and Histopathological Evaluations

**DOI:** 10.1002/vms3.70770

**Published:** 2026-01-06

**Authors:** Fatih Hatipoglu, Ayday Cunusova, Nariste Kadiraliyeva, Nur Abdimanap Uulu, Burak Mete, Orhan Corum, Kamil Uney

**Affiliations:** ^1^ Department of Pathology Faculty of Veterinary Medicine Selcuk University Konya Türkiye; ^2^ Faculty of Veterinary Medicine Kyrgyz‐Turkish Manas University Bishkek Kyrgyzstan; ^3^ Department of Pharmacology‐Toxicology Faculty of Veterinary Medicine, Selcuk University Konya Türkiye; ^4^ Department of Pharmacology‐Toxicology Faculty of Veterinary Medicine Hatay Mustafa Kemal University Hatay Türkiye

**Keywords:** biochemistry, haemogram, histopathology, quail, tolfenamic acid

## Abstract

**Objective:**

This study evaluated the safety profile of tolfenamic acid following repeated oral administration in Japanese quails (*Coturnix coturnix japonica*) at doses of 2 and 8 mg/kg every 12 h for 7 days.

**Methods:**

The 42 quails were randomly assigned to three groups. The first group (*n* = 6) received saline orally every 12 h for 7 days. The second (*n* = 18) and third (*n* = 18) groups were administered tolfenamic acid at doses of 2 and 8 mg/kg, respectively, orally every 12 h for 7 days, totalling 14 doses. The safety profile of tolfenamic acid was evaluated by haematological, biochemical and histopathological parameters.

**Results:**

Haematological analysis revealed no significant differences across groups, except for a decreased mean corpuscular haemoglobin concentration in the 8 mg/kg group. Biochemical assessments indicated stable liver and kidney function markers, as no significant changes were observed in alanine aminotransferase, aspartate aminotransferase, total cholesterol, albumin or creatinine levels. However, histopathological examinations showed significant liver changes, including hydropic degeneration and bile duct proliferation, as well as renal tubular epithelial degeneration, particularly in the higher dose group. Notably, lymphoid tissue depletion was observed in the spleen of treated birds.

**Conclusion:**

These findings indicate that tolfenamic acid administration does not adversely affect haematological or biochemical parameters. Although biochemical parameters remained normal, histopathological changes such as tissue damage may indicate early or subclinical injury that could impair organ function over time. These microscopic alterations might lead to long‐term health issues in quails, even without biochemical abnormalities. Therefore, cautious dosing and regular tissue monitoring are important when using tolfenamic acid.

## Graphical abstract

Safety of tolfenamic acid in Japanese quails.

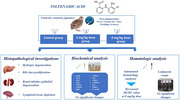

## 1 Introduction

Quails, like chickens, belong to the Galliformes order and the Phasianidae family, and they were first domesticated in Japan in 1595. Progress in quail production has been hindered by several management factors as well as infectious and non‐infectious diseases. Infectious diseases are particularly common among quail raised in intensive production systems. Since quail are closely related to poultry, many diseases affecting quail are like those affecting chickens and turkeys (El‐Ghany 2019).

Non‐steroidal anti‐inflammatory drugs (NSAIDs) are widely used in the treatment of inflammatory musculoskeletal diseases in both humans and animals. NSAIDs work by inhibiting cyclooxygenase (COX) activity, thereby reducing prostaglandin synthesis; however, information on their efficacy and optimal dosage in avian species is limited (Hadipour et al. 2011). Tolfenamic acid, or *N*‐(2‐methyl‐3‐chlorophenyl)‐anthranilic acid, is an NSAID in the phenamate group, structurally related to meclofenamic acid. Tolfenamic acid was initially developed for human use and has since been adopted in veterinary medicine. Tolfenamic acid is approved for use in veterinary medicine for the treatment of mastitis and respiratory tract infections in cattle, metritis‐mastitis‐agalactia syndrome in pigs, and postoperative analgesia in cats and dogs due to its analgesic, antipyretic and anti‐inflammatory effects (Landoni et al. 1996). Apart from its antipyretic effect, the pharmacodynamic effects of tolfenamic acid are dose‐dependent. In calves and dogs, tolfenamic acid's effects on body and skin temperature at doses of 2, 4 and 8 mg/kg are dose‐independent, while its effects on prostaglandin E_2_, thromboxane B_2_ (TxB_2_) and leucotrienes increase with dose. A 2 mg/kg dose of tolfenamic acid is recommended for an analgesic effect, whereas higher doses are suggested for an anti‐inflammatory effect. Tolfenamic acid exhibits dose‐dependent linear pharmacokinetics and does not have significant side effects on haematological parameters (Lees et al. 1998; McKellar et al. 1994).

Studies on tolfenamic acid in veterinary medicine have been conducted in sheep (Corum et al. 2018; Corum et al. 2024a), goats (Tekeli et al. 2020; Turk et al. 2021a), calves (Dinakaran and Dumka 2013), horses (Jaussaud et al. 1992), dogs (McKellar et al. 1994), rats (Patel et al. 2011), camels (Wasfi et al. 1998), geese (Turk et al. 2021b), turtles (Corum et al. 2019) and fish (Corum et al. 2024b). Although the pharmacokinetics of tolfenamic acid have been demonstrated in Japanese quails (Turk et al. 2021c), there is no information on the safety of different doses. Tolfenamic acid did not cause any clinical side effects after single‐dose administration of 2 mg/kg to quail (Turk et al. 2021c), ducks (Durna Corum et al. 2025) and partridges (Cetin et al. 2022). In addition, intravenous administration of tolfenamic acid to ducks at doses of 2, 4 and 8 mg/kg was well tolerated and did not cause significant adverse effects on biochemical parameters (Corum et al. 2024c). Bird species are more susceptible than mammals to the adverse effects of NSAIDs, including renal ischaemia and tissue damage, and adverse effects may vary among species (Toutain et al. 2010). Therefore, establishing the safety of tolfenamic acid in quails is of clinical importance. Given that high doses or repeated use of NSAIDs have adverse effects on the kidney, liver and haematological systems, and that birds are more susceptible to these effects, it was hypothesized that the safety of repeated doses of 2 and 8 mg/kg tolfenamic acid in quails should be determined. This study aims to determine the haematological, biochemical and pathological effects of tolfenamic acid after repeated oral administration (2 or 8 mg/kg, every 12 h for 7 days) at different doses in Japanese quail.

## 2 Material and Methods

### 2.1 Experimental Animals

This study used 42 Japanese quails (*Coturnix coturnix japonica*) aged 60–70 days, with an average body weight of 210 ± 15 g, obtained from the Quail Unit at the Faculty of Veterinary Medicine, Kyrgyz‐Turkish Manas University. The quails were housed in cleanable wire cages with six birds per cage, under controlled conditions of 24 ± 1°C temperature and 60% humidity, with a 12‐h light/12‐h dark cycle. Health checks were conducted prior to the study, and the quails were acclimatized for 2 weeks. Feed and water were provided ad libitum. The feed composition included corn, soybean, wheat, meat and bone meal, wheat bran, soybean meal and a vitamin–mineral mixture, providing 3000 kcal/kg of metabolic energy, 20% crude protein and 4.4% crude fibre. All procedures were approved by the Ethics Committee of the Faculty of Veterinary Medicine, Kyrgyz‐Turkish Manas University (Approval No. 2021/03, dated 28 October 2021).

### 2.2 Experimental Groups and Drug Administration

Tolfenamic acid was approved for use in mammals at doses of 2–4 mg/kg (Corum et al. 2024a). It has been used at doses of 2–8 mg/kg in studies conducted in various avian species (Corum et al. 2024c; Turk et al. 2021b, 2021c). When the efficacy of 2, 4 and 8 mg/kg doses of tolfenamic acid was compared in dogs and calves, it was stated that the effect was dose‐dependent and that higher doses showed better efficacy and did not cause adverse effects. Therefore, the use of 2 and 8 mg/kg doses was preferred in this study.

The 42 quails were randomly assigned to three groups. The first group (*n* = 6) received saline orally every 12 h for 7 days. The second (*n* = 18) and third (*n* = 18) groups were administered tolfenamic acid at doses of 2 and 8 mg/kg, respectively, orally every 12 h for 7 days, totalling 14 doses. All drug administrations were performed without food restriction. The animals were monitored twice daily after each dose for changes in respiration, circulation, autonomic and central nervous system functions, skin condition, feather quality, eye and mucous membrane appearance, and behavioural responses. Feed and water consumption per cage (6 quails per cage) was recorded twice daily. Body weights were measured before the first dose and after the final dose. Twelve hours after the last tolfenamic acid dose, blood samples were collected from the right or left brachial veins into K3 EDTA tubes (∼0.5 mL) and gel tubes (∼1 mL) using 26‐gauge insulin syringes. The quails were then sacrificed by decapitation for tissue sampling.

### 2.3 Haematological Analysis

Blood samples collected in EDTA tubes were used for haematological analysis, which was conducted within 1 h of sample collection. Parameters measured included red blood cell (RBC), haemoglobin (HGB), haematocrit (HCT), mean corpuscular volume (MCV), mean corpuscular haemoglobin (MCH), mean corpuscular haemoglobin concentration (MCHC) and platelets (PLT), using an automated haematology analyser (Auto Hematology Analyzer, BC‐2300, Mindray).

### 2.4 Leucocyte Count

To count leucocyte types, blood smears were prepared by placing a drop of EDTA‐treated blood on a slide, air‐drying it and staining it using the JorVet Dip Quick rapid stain set (Jorgensen Laboratories Inc.). Differential leucocyte counts [basophils (BASO), eosinophils (EOS), monocytes (MONO), band neutrophils (BN), segmented neutrophils (SN) and lymphocytes (LYM)] were performed under a light microscope at 100x magnification with immersion oil. Counts were recorded until 100 leucocytes were observed, and the percentages of each leucocyte type were calculated using a manual differential count.

### 2.5 Biochemical Analysis

For biochemical analysis, blood samples in gel tubes were centrifuged at 4000 g for 10 min. The resulting serum samples were stored at −80°C until analysis. Using an autoanalyser (ARCHITECT C8000, Abbott Diagnostics), serum levels of albumin (ALB), alkaline phosphatase (ALP), alanine aminotransferase (ALT), aspartate aminotransferase (AST), cholesterol (CHOL), direct bilirubin (D‐BLB), high‐density lipoprotein (HDL) cholesterol, total bilirubin (T‐BLB), creatine kinase (CK), creatinine (CREA), total protein (T‐PROT), triglyceride (TG), urea (UREA) and uric acid (U‐ACID) were measured.

### 2.6 Pathological Investigations

After the quails were sacrificed, a systemic necropsy was performed, and the liver, kidney and spleen were removed. These organs were then cleaned of blood, weighed, and their relative organ weights were calculated. Photographs were taken of cases where macroscopic changes were observed. Colour changes detected in the liver in macroscopic examinations were evaluated subjectively. For histopathological examination, tissue samples were fixed in 10% buffered formalin. Following routine processing, paraffin blocks were prepared, and 5‐µm‐thick sections were obtained using a microtome (Leica, RM2255). All sections were stained with haematoxylin and eosin (H&E) (Luna 1968). Each preparation was examined under a light microscope, and microscopic images were captured as necessary (Euromex, IS.1153.EPL). Lesions in the organs were generally graded on a scale: healthy (0), mild (1), moderate (2) and severe (3). Microscopically, hepatocellular degeneration in livers was graded as follows: healthy (0): no change, mild (1): mild hepatocellular swelling due to hydropic degeneration and fatty changes, moderate (2): clear hepatocellular swelling due to hydropic degeneration and fatty changes and severe (3): diffuse and severe hepatocellular swelling, cytoplasmic paleness and rupture. Tubular degeneration in kidneys was graded as follows: healthy (0): no change, mild (1): mild tubular epithelial swelling due to hydropic degeneration, moderate (2): clear tubular epithelial swelling due to hydropic and ballooning degeneration and severe (3): diffuse and severe tubular epithelial swelling, cytoplasmic paleness and rupture. Lymphoid tissue depletion in spleen was graded as follows: healthy (0): no change, mild (1): small amount of lymphoid tissue depletion less than 30%, moderate (2): moderate lymphoid tissue depletion of 30%–60% and severe (3): a significant depletion of lymphoid tissue, more than 60%.

### 2.7 Statistical Analysis

All data were presented as mean ± SD (standard deviation). The Shapiro–Wilk and Levene's tests were used to assess normality and homogeneity of the data distribution. Statistical analyses were performed using SPSS 22.0 (IBM Corp., Armonk, NY). A 95% confidence interval was applied to all analyses, with statistical significance set at *p* < 0.05. Differences between groups in haemogram, biochemistry, body and organ weights were analysed using one‐way analysis of variance (ANOVA), with Tukey's test for post hoc comparisons. Data on liver colour changes (number of quails showing changes/total number of quails examined) and histopathological data were evaluated using the chi‐square test. The weighted mean score of degenerations in the liver, kidney and spleen was assessed by one‐way ANOVA followed by post hoc Duncan's test, with data presented as mean ± SE.

## 3 Results

No systemic effects of tolfenamic acid on the respiratory, circulatory, autonomic or central nervous systems were observed in the skin, feather quality, eye and mucous membrane appearance, somatomotor activity or behaviour observations made on quails following drug administration.

The haemogram parameters of tolfenamic acid following repeated oral administration at different doses in Japanese quails are presented in Table 1. When comparing the study groups, no statistically significant differences were observed in haematological parameters, except for MCHC (*p* > 0.05). However, the MCHC value was statistically lower in the 8 mg/kg dose group compared to the control group (*p* < 0.05). BASO and MONO values were significantly higher and lower, respectively, in the 8 mg/kg dose group compared to the 2 mg/kg dose group (*p* < 0.05).

**TABLE 1:** Haemogram parameters following oral administration of tolfenamic acid (every 12 h for 7 days) at doses of 2 and 8 mg/kg in Japanese quail.

Parameter	Control (*n* = 6)	2 mg/kg (*n* = 18)	8 mg/kg (*n* = 18)
RBC (×10^12^/L)	3.37 ± 0.38	3.286 ± 0.305	3.213 ± 0.353
HGB (g/L)	171.67 ± 24.05	168.389 ± 14.657	163.500 ± 16.133
HCT (%)	45.617 ± 5.746	45.667 ± 3.742	45.278 ± 4.824
MCV (fL)	135.233 ± 6.256	138.906 ± 5.681	140.694 ± 4.878
MCH (pg)	50.817 ± 2.134	51.339 ± 2.556	51.006 ± 2.171
MCHC (g/L)	375.900 ± 9.992^a^	369.528 ± 11.714^ab^	362.556 ± 9.522^b^
PLT (×10^9^/L)	12.333 ± 3.830	11.556 ± 2.791	13.833 ± 3.073
BN (%)	5.667 ± 2.422	7.500 ± 3.348	6.556 ± 3.698
SN (%)	49.500 ± 7.740	46.444 ± 9.805	47.778 ± 8.748
LY (%)	27.000 ± 6.723	29.944 ± 6.760	31.389 ± 8.346
BASO (%)	3.333 ± 2.805^ab^	2.444 ± 1.338^b^	6.889 ± 5.603^a^
EOS (%)	4.000 ± 2.000	3.222 ± 2.881	3.389 ± 4.552
MONO (%)	6.167 ± 4.167^ab^	10.444 ± 4.718^a^	4.000 ± 3.896^b^

*Note*: Data are presented as mean ± SD. Values within a row marked with different letters (a, b) are significantly different (*p* < 0.05).

Abbreviations: BASO, basophil; BN, band neutrophil; EOS, eosinophil; HCT, haematocrit; HGB, haemoglobin; LY, lymphocyte; MCH, mean corpuscular haemoglobin; MCHC, mean corpuscular haemoglobin concentration; MCV, mean corpuscular volume; MONO, monocyte; PLT, platelet; RBC, red blood cell; SN, segmented neutrophil.

The serum biochemistry parameters following oral administration of tolfenamic acid at different doses in Japanese quails are presented in Table 2. There were no statistically significant differences in the biochemical parameters when comparing the study groups (*p* > 0.05).

**TABLE 2:** Serum biochemistry parameters following oral administration of tolfenamic acid (every 12 h for 7 days) at different doses in Japanese quail.

Parameter	Control (*n* = 6)	2 mg/kg (*n* = 18)	8 mg/kg (*n* = 18)
ALB (g/dL)	0.883 ± 0.117	0.939 ± 0.311	0.967 ± 0.247
ALP (U/L)	265.833 ± 67.783	280.056 ± 66.755	289.056 ± 81.019
ALT (U/L)	9.167 ± 2.994	10.944 ± 5.589	9.667 ± 3.850
AST (U/L)	156.167 ± 41.653	175.444 ± 49.149	184.667 ± 50.452
CHOL (mg/dL)	100.67 ± 13.261	105.167 ± 25.762	122.889 ± 33.134
D‐BLB (mg/dL)	0.108 ± 0.045	0.132 ± 0.088	0.13 ± 0.06
HDL (mg/dL)	42.17 ± 14.13	43.83 ± 14.76	53.33 ± 17.42
T‐BLB (mg/dL)	0.24 ± 0.08	0.29 ± 0.18	0.30 ± 0.13
CK (U/L)	398 ± 40	403 ± 50	411 ± 80
CREA (mg/dL)	0.21 ± 0.02	0.206 ± 0.015	0.202 ± 0.007
T‐PROT (g/dL)	4.23 ± 0.43	4.400 ± 0.840	4.356 ± 0.631
TG (mg/dL)	496 ± 241	398 ± 160	356 ± 239
UREA (mg/dL)	3.67 ± 1.03	3.556 ± 1.149	3.667 ± 1.188
U‐ACID (mg/dL)	2.97 ± 2.04	3.456 ± 1.162	4.311 ± 1.118

*Note*: Data are presented as mean ± SD. No statistically significant differences were observed between groups (*p* > 0.05).

Abbreviations: ALB, albumin; ALP, alkaline phosphatase; ALT, alanine aminotransferase; AST, aspartate aminotransferase; CHOL, cholesterol; CK, creatine kinase; CREA, creatinine; D‐BLB, direct bilirubin; HDL cholesterol, high‐density lipoprotein cholesterol; T‐BLB, total bilirubin; TG, triglyceride; T‐PROT, total protein; U‐ACID, uric acid; UREA, urea.

**FIGURE 1:**
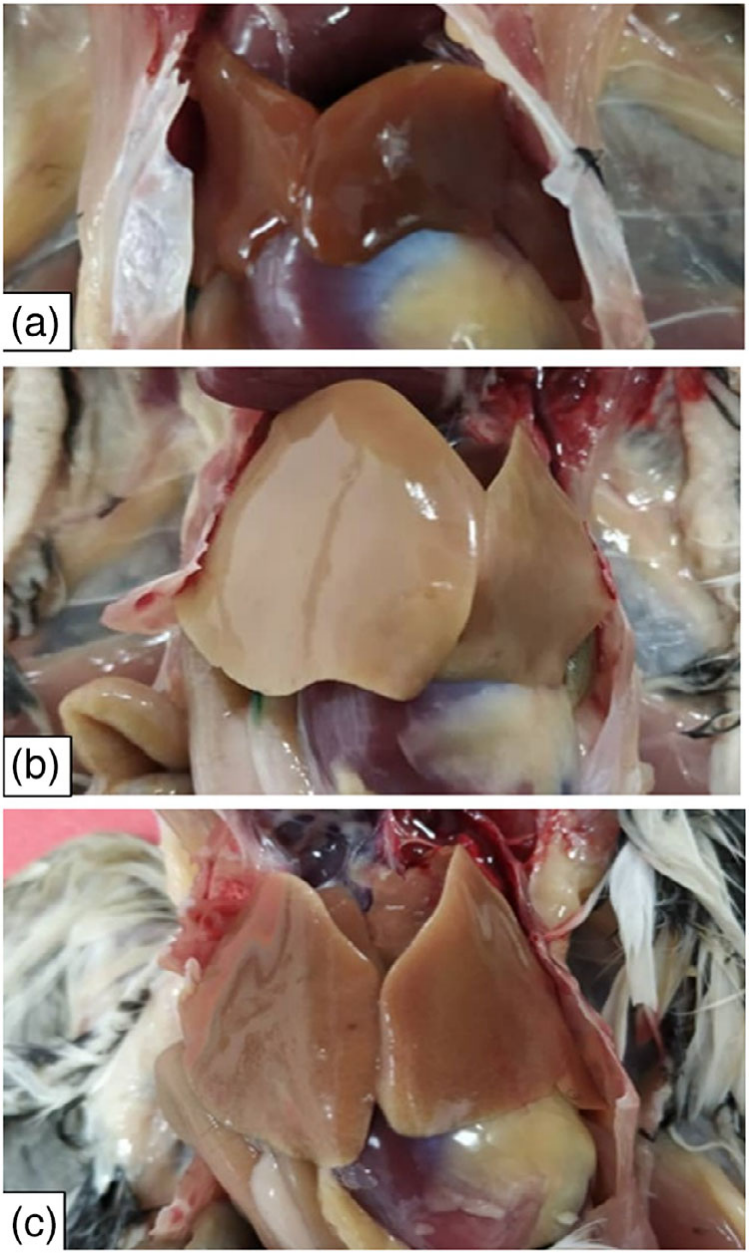
Liver. (A) Normal (control), (B) severe paleness (2 mg/kg) and (C) moderate paleness (8 mg/kg).

The body and organ weights of Japanese quails following oral administration of tolfenamic acid at different doses are presented in Table 3. Japanese quails were euthanized following oral administration of tolfenamic acid at different doses, and both macroscopic and histopathological changes in the organs were evaluated after necropsy. No statistically significant difference was detected in terms of body and organ weights among the groups. (*p* > 0.05) (Table 3). Liver colour changes were evaluated by chi‐square testing. Liver paleness occurred in 2/6 (33.3%) control Japanese quails, 15/18 (83.3%) in the 2 mg/kg group and 16/18 (88.9%) in the 8 mg/kg group (*χ* ^2^(2, *N* = 42) = 11.32, *p* = 0.0035). Both treated groups showed significantly higher paleness incidence than controls (Table 3). It was observed that the livers in the 2 and 8 mg/kg groups were moderately and severely pale, respectively, compared to the control group (Figure 1).

**TABLE 3:** Body and organ weights and liver colour changes of Japanese quails following oral administration of tolfenamic acid (every 12 h for 7 days) at doses of 2 and 8 mg/kg.

Parameter	Control (*n* = 6)	2 mg/kg (*n* = 18)	8 mg/kg (*n* = 18)
Body and organ weights^*^			
Live weight (g)	206.67 ± 19.41	206.11 ± 25.93	216.94 ± 20.30
Liver (g)	3.583 ± 1.080	4.961 ± 1.437	5.201 ± 1.600
*Relative liver*	*1.760 ± 0.572*	*2.384 ± 0.588*	*2.382 ± 0.617*
Spleen (g)	0.110 ± 0.111	0.177 ± 0.205	0.165 ± 0.057
*Relative spleen*	*0.054 ± 0.052*	*0.086 ± 0.097*	*0.076 ± 0.025*
Kidney (g)	1.322 ± 0.244	1.493 ± 0.328	1.638 ± 0.353
*Relative kidney*	*0.644 ± 0.136*	*0.722 ± 0.126*	*0.751 ± 0.124*
Liver colour changes^**^			
Liver paleness	2/6^a^	15/18^b^	16/18^b^
*Mild*	*2*	*2*	*5*
*Moderate*	*—*	*6*	*6*
*Severe*	*—*	*7*	*5*

* Data were given as mean ± SD.

** Data were given as number of quails showing changes/total number of quails. In addition, within the same row of the data, different letters (a, b) indicate statistically significant differences (*p* < 0.05).

**FIGURE 2:**
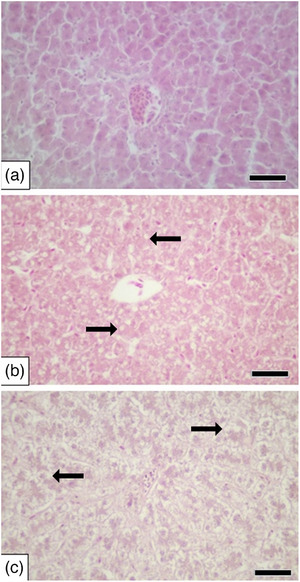
Liver. (A) Normal microscopic appearance of the liver (control), H&E, scale bar: 50 µm, (B) hepatocellular hydropic degeneration/fatty changes (arrows) (moderate) (2 mg/kg), H&E, scale bar: 50 µm and (C) hepatocellular hydropic degeneration/fatty changes (arrows) (severe) (8 mg/kg), H&E, scale bar: 50 µm.

**FIGURE 3:**
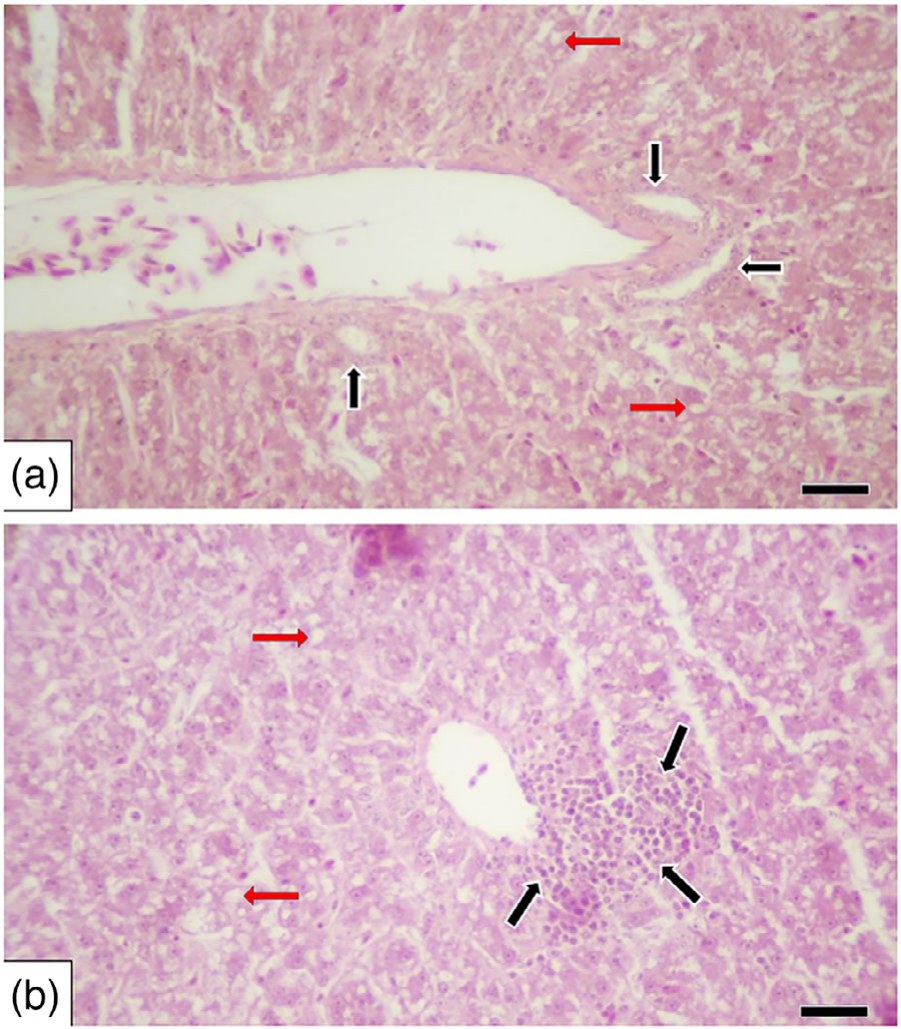
Liver. (A) Hepatocellular hydropic degeneration/fatty changes (red arrows) and increased number of bile ducts (black arrows), (2 mg/kg), H&E, scale bar: 50 µm and (B) hepatocellular hydropic degeneration/fatty changes (red arrows) and mononuclear cell infiltration in the portal area (black arrows), (8 mg/kg), H&E, scale bar: 50 µm.

As a result of histopathological examinations (Table 4), hydropic degeneration/fatty changes were detected in 1/6 (16.7%) Japanese quails in the control group, whereas both the 2 and 8 mg/kg groups showed 18/18 (100%) affected Japanese quails. Chi‐square analysis confirmed a highly significant difference among groups (*χ* ^2^(2, *N* = 42) = 59.19; *p* = 4.03 × 10^−8^). This change was moderately more common in the 2 mg/kg group, while it was mildly and moderately more prevalent in the 8 mg/kg group (Table 4 and Figure 2). In addition, bile duct proliferation in the portal area was not observed in the control group, but it was noted in three quails in the 2 mg/kg group and in one quail in the 8 mg/kg group (Table 4 and Figure 3A). Furthermore, while mononuclear cell infiltration in the portal area was absent in the control group, it was observed in two cases in the 2 mg/kg group and in one case in the 8 mg/kg group (Table 4 and Figure 3B). However, there was no statistically significant difference between the groups in terms of bile duct proliferation and mononuclear cell infiltration in the portal area (*p* > 0.05) (Table 4).

**TABLE 4:** Histopathological changes in the liver, kidney and spleen following oral administration (every 12 h for 7 days) of tolfenamic acid at doses of 2 and 8 mg/kg in Japanese quail.

	Parameter	Control (*n* = 6)	2 mg/kg (*n* = 18)	8 mg/kg (*n* = 18)
Liver				
	Hydropic degeneration/fatty changes	1/6^a^	18/18^b^	18/18^b^
	*Mild*	*1*	*4*	*7*
	*Moderate*	*—*	*11*	*7*
	*Severe*	*—*	*3*	*4*
	Total weighted liver lesion score	0,17^a^ ± 0,40^a^	1,95 ± 0,64^b^	1,83 ± 0,79^b^
	Bile duct proliferation	‐/6	3/18	1/18
	Mononuclear cell infiltration in the portal area	‐/6	2/18	1/18
Kidney				
	Degeneration in tubule epithelium	1/6^a^	14/18^b^	15/18^b^
	*Mild*	*1*	*12*	*12*
	*Moderate*	*—*	*2*	*3*
	*Severe*	*—*	*—*	*—*
	Total weighted kidney lesion score	0.17 ± 0.40^a^	1.55 ± 0.85^b^	1.50 ± 0.79^b^
Spleen				
	Lymphoid tissue depletion	‐/6	2/18	3/18
	*Mild*	*—*	*2*	*3*
	*Moderate*	*—*	*—*	*—*
	*Severe*	*—*	*—*	*—*
	Total weighted spleen lesion score	0.00 ± 0.00	0.11 ± 0.32	0.16 ± 0.39

*Note*: Different letters (a, b) in the same row indicate statistically significant differences (*p* < 0.05).

**FIGURE 4:**
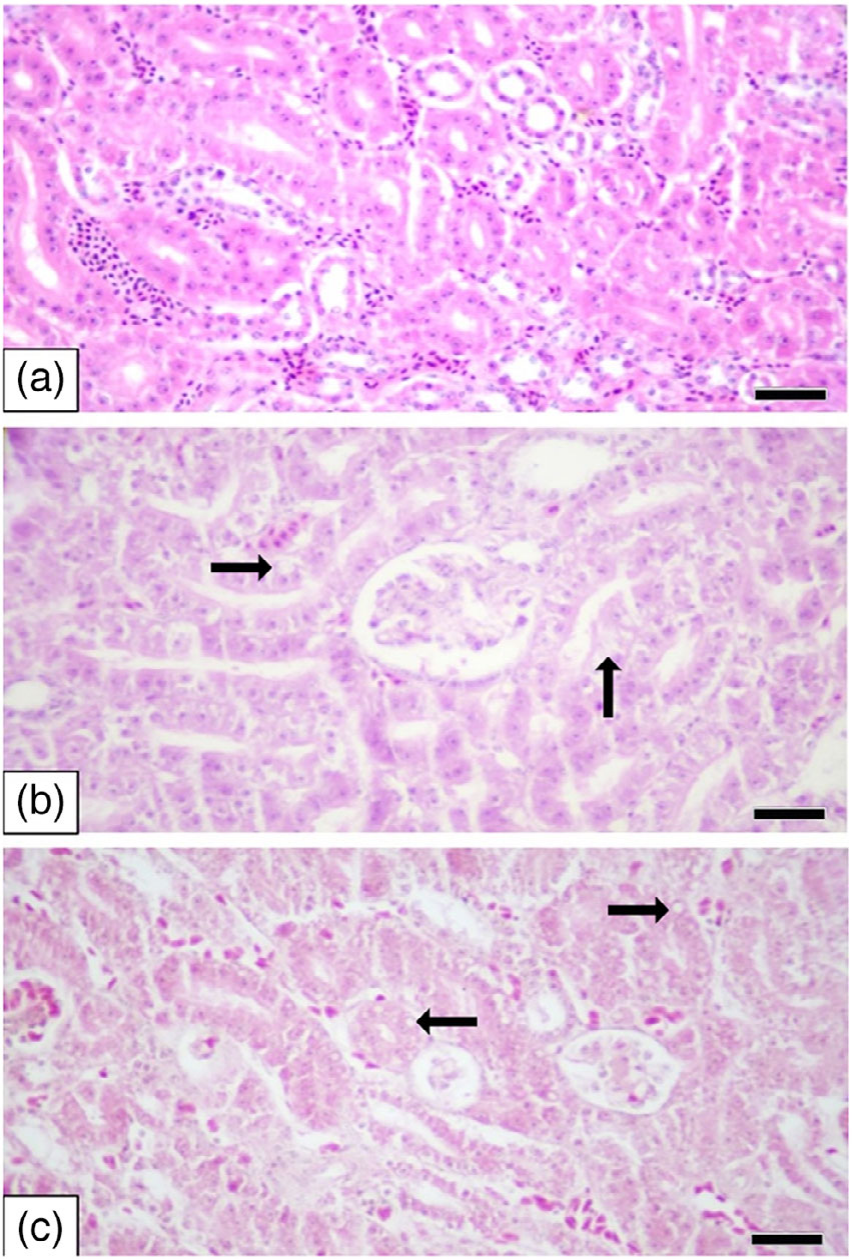
Kidney. (A) Normal microscopic view of the kidney (control), H&E, scale bar: 50 µm, (B) tubule epithelial degeneration (arrows) (mild) (2 mg/kg), H&E, scale bar: 50 µm and (C) tubule epithelial degeneration and vacuolization (arrows) (moderate) (8 mg/kg), H&E, scale bar: 50 µm.

As a result of the histopathological examinations of the kidneys, tubule epithelial degeneration was observed in 1 case (mild) in the control group, 14 cases (12 mild, 2 moderate) in the 2 mg/kg group, and 15 cases (12 mild, 3 moderate) in the 8 mg/kg group (Figure 4). Chi‐square analysis demonstrated a significant difference in incidence across groups (*χ* ^2^(2, *N* = 42) = 10.42; *p* = 0.00546). Mean ± SE total weighted kidney lesion scores were 0.17 ± 0.40 in control, 1.55 ± 0.85 in the 2 mg/kg group and 1.50 ± 0.79 in the 8 mg/kg group. One‐way ANOVA revealed a significant difference among groups (*F*(2, 39) = 7.34; *p* = 0.0021) (Table 4).

**FIGURE 5:**
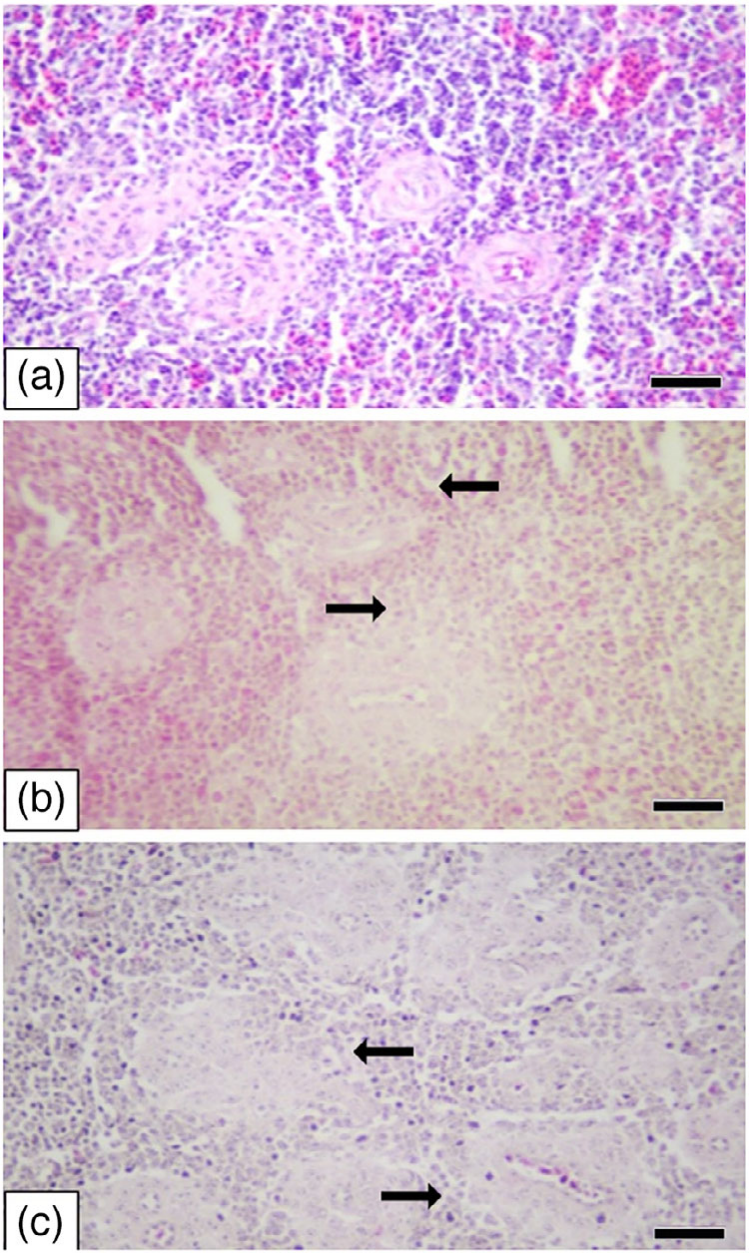
Spleen. (A) Normal microscopic appearance of the spleen (control), H&E, scale bar: 50 µm, (B) lymphoid tissue depletion (arrows) (mild) (2 mg/kg), H&E, scale bar: 50 µm and (C) lymphoid tissue depletion (arrows) (mild) (8 mg/kg), H&E, scale bar: 50 µm.

As a result of the histopathological examinations of the spleen, lymphoid tissue depletion was not found in the control group; however, it was observed in two cases (mild) in the 2 mg/kg group and in three cases (mild) in the 8 mg/kg group (Figure 5).

Chi‐square test showed no significant difference among groups (*χ* ^2^(2, *N* = 42) = 1.21; *p* = 0.5459).

## 4 Discussion

In this study, the effects of repeated (every 12 h for 7 days) administration of tolfenamic acid at doses of 2 and 8 mg/kg on haematological, biochemical and pathological parameters were demonstrated for the first time. Tolfenamic acid is commonly used in mammals and reptiles at doses of 2–4 mg/kg (Corum et al. 2018; Corum et al. 2019; Tekeli et al. 2020), and prior reports indicate its use in quails at 2 mg/kg (Turk et al. 2021a). Given that anti‐inflammatory effects are dose‐dependent (McKellar et al. 1994), we evaluated doses of 2 and 8 mg/kg, with the latter being double the typical upper dose of 4 mg/kg. Oral administration was employed, as it is the preferred route for quails (Miller et al. 2019).

Birds are notably vulnerable to renal toxicity from NSAIDs, and safety profiles can differ significantly across species (Hussain et al. 2008). NSAIDs like diclofenac and flunixin have been linked to declining vulture populations (Zorrilla et al. 2015). Side effects from NSAIDs, such as gastrointestinal tissues and kidney damage, are often due to COX‐1 inhibition (Zollinger et al. 2011), while COX‐2 inhibition may also contribute to renal toxicity (Brater et al. 2001; Harris 2006). In contrast, meloxicam, with selective COX‐2 activity, showed no significant adverse effects in quails at 2 mg/kg IM every 12 h (Sinclair et al. 2012). Tolfenamic acid has been reported to be safe in vultures (Galligan et al. 2020), and the risk of gastrointestinal bleeding in humans and rats is lower than other NSAIDs (Eskerod 1994; Hendel 1994). The inhibitory impact of tolfenamic acid on COX enzymes in birds remains undetermined, whereas it is non‐selective in calves (Landoni et al. 1996) and particularly effective on COX‐2 enzymes in goats (Sidhu et al. 2006). Since tolfenamic acid is generally safe in birds, it may be particularly effective on COX‐2 enzymes, but this information needs to be investigated. Studies indicate no changes in haematological, biochemical or histopathological parameters in rats at 4 mg/kg for 14 days (Patel et al. 2011), no haematological and biochemical parameters in sheep at 2–8 mg/kg IV and no haematological parameters in dogs at 2–8 mg/kg IM, respectively (McKellar et al. 1994; Yildiz et al. 2019; Corum et al. 2023). However, tolfenamic acid did not cause any effect on biochemical parameters except ALT in ducks when administered intravenously at doses of 2–8 mg/kg (Corum et al. 2024c).

The haematopoietic system serves as a crucial indicator of physiological and pathological conditions in both humans and animals, making it one of the most sensitive systems to toxic substances (Li et al. 2010). In addition, it provides significant insights into intravascular effects, such as bone marrow activity and haemolysis (Vishnu et al. 2010). In this study, haematological parameters, with the exception of MCHC, were comparable across the groups. Notably, the MCHC value was significantly lower in the 8 mg/kg dose group compared to the control group. MCHC is calculated from HGB and HCT levels. In this study, there was an insignificant decrease in HGB and RBC in the 8 mg/kg dose group. NSAID use may lead to gastrointestinal bleeding and iron deficiency, which can result in reduced HGB levels (Goldstein et al. 2011). In addition, it has been reported that some NSAIDs such as mefenamic acid, ibuprofen, sulindac, naproxen, tolmetin, feprazone and aspirin can cause haemolysis, leading to decreases in both RBC and HGB levels (Barbaryan et al. 2013; Sanford‐Driscoll and Knodel 1986). The reduction in MCHC observed in the 8 mg/kg dose group may be attributed to iron deficiency secondary to gastrointestinal bleeding and/or subclinical haemolysis induced by this dose. Regarding the BASO and MONO parameters, while the dose groups remained similar to the control group, the 8 mg/kg group exhibited a higher percentage of BASO, and a lower percentage of MONO compared to the 2 mg/kg group. Overall, the haematological results indicate that oral administration of tolfenamic acid at doses of 2 and 8 mg/kg, given twice daily for 7 days, did not produce any toxic effects on the haematopoietic system.

In this study, the ALT and AST values were comparable between the groups when the 2 and 8 mg/kg doses of tolfenamic acid were compared to the control group. ALT and AST are established markers for liver function and are commonly used to detect toxic effects (El Hilaly et al. 2004). Damage to liver parenchymal cells typically leads to elevated levels of these transaminases. Notably, serum AST levels originate from both mitochondrial and cytoplasmic sources and are primarily elevated in the presence of cellular damage. Total CHOL is also considered an indirect marker of liver function (El Hilaly et al. 2004). In our study, no significant differences in CHOL levels were observed in the 2 and 8 mg/kg doses of tolfenamic acid compared to the control group. The liver is responsible for the synthesis and degradation of most plasma proteins; therefore, decreased plasma protein levels may indicate increased catabolism and/or impaired synthesis within hepatocytes due to various physiological and pathological events (Killingsworth 1979; Johnson 1999). In this study, no significant differences were found in ALB and T‐PROT levels.

CREA is a reliable marker of renal function, with serum CREA levels increasing in response to nephron damage (Lameire et al. 2005). The administration of tolfenamic acid did not alter CREA levels compared to the control group. However, histopathological examinations of liver and kidney tissues revealed that the total weighted lesion scores for both organs were higher in the 2 and 8 mg/kg dose groups than in the control group. This suggests that tolfenamic acid may have toxic effects on the liver and kidney at both dose levels. Interestingly, despite the observed histopathological changes in liver and kidney tissues, no significant differences were found in biochemical parameters indicative of organ function when compared to the control group. The reasons for these discordant results are not entirely understood but are likely multifactorial. They may stem from variations in tissue damage and repair mechanisms, organ adaptation, time lag in biomarker release, the poor sensitivity and specificity of these traditional markers, animal health status, nutritional factors and age (Travlos et al. 1996). Previous toxicity studies have indicated that the positive correlation between biochemical parameters and histopathological findings can vary over time, with increased exposure duration enhancing the correlation rate (Travlos et al. 1996). For instance, hepatocellular vacuolization was found to increase in a dose‐dependent manner following meloxicam administration in American kestrels, yet T‐BLB, D‐BLB and ALB levels remained unchanged, and AST values decreased after 7 days of treatment at varying doses (2–20 mg/kg, every 12 h for 7 days) (Summa et al. 2017).

The long‐term use of tolfenamic acid can lead to serious side effects, including liver and gastrointestinal damage (Monteiro‐Steagall et al. 2013). No statistically significant difference was detected in absolute and relative liver weights. When evaluating colour changes in the liver, a significant difference was noted between the control group and both the 2 and 8 mg/kg groups. Livers from the 2 and 8 mg/kg groups appeared moderately and severely pale, respectively, compared to the control group. Due to the lack of existing studies examining relative liver weights and colour changes following tolfenamic acid administration in quails, direct comparisons could not be made. Sankpal et al. (2013) treated mice with 50 mg/kg tolfenamic acid three times a week for 6 weeks and found no differences in the cellular characteristics of intestinal, liver and stomach tissues between control and tolfenamic acid‐treated groups upon microscopic examination at the study's conclusion. Conversely, Zhou et al. (2020) reported lymphocytic infiltration in the portal area, dilation and congestion in sinusoids, binucleation of hepatocytes and an increase in Kupffer cells in the liver tissue of mice after oral administration of tolfenamic acid at a dose of 100 mg/kg once daily for 6 days.

In addition, Sinclair et al. (2012) found that lymphocytic hepatitis and hepatic lipidosis were observed in the liver following intramuscular administration of meloxicam at a dose of 2 mg/kg for 14 days (administered every 12 h) in quails. Hepatitis was significantly more frequent in the meloxicam‐treated group compared to the control group, while no significant difference in the severity of hepatic lipidosis was noted between groups. Furthermore, another study reported that carprofen administered to pigeons at doses of 2, 5 and 10 mg/kg/day for 7 days via the intramuscular route resulted in diffuse lipidosis, hydropic degeneration and hepatitis in hepatocytes observed at various intervals (2‐, 4‐, 6‐ and 8‐day post‐administration) (Zollinger et al. 2011). In the present study, histopathological examinations revealed a statistically significant difference in hydropic degeneration/fatty changes in the liver between the control group and both the 2 and 8 mg/kg groups. This change was observed to be moderately more common in the 2 mg/kg group, while it was reported as mildly and moderately more common in the 8 mg/kg group. Bile duct proliferation in the portal area was not detected in the control group; however, it was observed in three cases in the 2 mg/kg group and in one case in the 8 mg/kg group. Similarly, mononuclear cell infiltration in the portal area was absent in the control group but identified in two cases in the 2 mg/kg group and in one case in the 8 mg/kg group. Despite these findings, there was no statistically significant difference between the groups regarding bile duct proliferation and mononuclear cell infiltration in the portal area. While there are studies examining NSAID‐related histopathological changes in various animals (Sankpal et al. 2013; Sinclair et al. 2012; Zhou et al. 2020; Zollinger et al. 2011), a direct comparison could not be made due to the absence of research specifically addressing histopathological changes in the liver following tolfenamic acid administration in quails.

Regarding kidney histopathology, no studies were found comparing the effects of tolfenamic acid administration. In this study, tubule epithelial degeneration was noted, with 1 mild case in the control group, 14 cases (12 mild, 2 moderate) in the 2 mg/kg group, and 15 cases (12 mild, 3 moderate) in the 8 mg/kg group. A statistically significant difference was observed between the control group and both the 2 and 8 mg/kg groups concerning tubule epithelial degeneration.

In a study (Palocz et al. 2016) investigating the pharmacotoxicological effects of diclofenac (5 and 50 mg/kg bw.) and acetylsalicylic acid (50 mg/kg bw) on four bird species, including broiler chickens, domestic pigeons, budgerigars and quails, both doses of diclofenac caused harmful effects in the examined species were reported. The researchers stated that the high‐dose diclofenac caused death in all four species; pigeon was the least sensitive to diclofenac, the acetylsalicylic acid was clinically well tolerated in all examined species. The researchers (Palocz et al. 2016) stated that they observed the following changes in histopathological examinations: following 50 mg/kg diclofenac administration, serious tubulonephrosis and marked tubular epithelial necrosis in the kidneys, and granulocyte infiltration, cytoplasmic vacuolization and some necrotic hepatocyte cells in the liver. In another study (Hussain et al. 2008) examining the toxicological effects of diclofenac on four avian species including broiler chicks, pigeons, Japanese quail and mynah; serum CREA levels were reported to be high in all species, while serum urea levels did not change significantly in broiler chickens, decreased significantly in pigeons and increased significantly in Japanese quail and mynah. The researchers declared that the kidneys and liver were enlarged in all species. Histologically, acute renal necrosis in the kidneys and fatty change and necrosis of hepatocytes in the liver were reported in all species (Hussain et al. 2008). In these studies (Hussain et al. 2008; Palocz et al. 2016), necrotic changes were reported in the kidney tubule epithelium and hepatocytes in the liver, but in the presented study, unlike these researchers, hydropic degeneration in the kidneys and hydropic degeneration/fatty change in the liver were observed in the tolfenamic acid groups. It was concluded that the reason for this difference in histopathological findings was due to the dose and duration of the drugs used.

It has been reported that spleen weights may increase due to factors such as leucocytosis, extramedullary haematopoiesis, erythrophagocytosis or septic shock (Zhou et al. 2020). In the present study, there were no statistically significant differences in both absolute and relative spleen weights between the control group and the tolfenamic acid‐treated groups (2 and 8 mg/kg). As there are no available studies examining relative spleen weights following tolfenamic acid administration in quails, no direct comparisons could be made. However, Zhou et al. (2020) noted that oral administration of tolfenamic acid at a dose of 100 mg/kg once daily for 6 days resulted in a significant increase in spleen index (Spleen index = spleen weight [mg]/body weight [g]) compared to the control group in their mouse study. In histopathological examinations of the spleen, lymphoid tissue depletion was observed in two cases (mild) in the 2 mg/kg group and in three cases (mild) in the 8 mg/kg group, but a statistically no significant difference between the control group and the tolfenamic acid‐treated groups. It has been stated that NSAIDs such as naproxen and indomethacin reduce LYM proliferation in the spleen by inhibiting T cell proliferation (Cicala et al. 2000). Depletion of lymphoid tissue in the spleen resulting from tolfenamic acid administration may be attributed to the suppression of T cell proliferation. In the present study, although lymphoid tissue depletion in the spleen was observed in both tolfenamic acid groups (mild degree), no statistically significant difference was found between the control group and tolfenamic acid‐treated groups, which may be due to the dose or duration of tolfenamic acid administered.

This study had some limitations. In this study, the effects of repeated tolfenamic administration (2 or 8 mg/kg every 12 h) for 7 days on haematological, biochemical and histopathological parameters were investigated. The possible adverse effects of tolfenamic acid were not investigated at the molecular level. In this study, the short‐term side effects of tolfenamic acid were investigated; longer‐term use is needed to evaluate its chronic effects.

## 5 Conclusion

We conclude that repeated oral administration of tolfenamic acid at doses of 2 and 8 mg/kg in Japanese quails has notable impacts on histopathological parameters. While haematological parameters remained largely unaffected, a statistically significant decrease in MCHC at the higher dose suggests potential changes in erythrocyte characteristics. Biochemical assessments indicated no significant alterations in liver and kidney function markers, yet histopathological evaluations revealed significant changes, including hydropic degeneration and epithelial damage in the liver and kidney tissues, particularly at the higher dose. The observed lymphoid tissue depletion within the spleen and tubule epithelial degeneration further underscores the need for caution when using tolfenamic acid, especially at elevated doses.

These findings underscore the necessity of comprehensive monitoring, including histopathological, biochemical and functional assessments, to fully understand the safety profile of tolfenamic acid in avian species. While current data reveal notable tissue changes at both tested doses, the mechanisms underlying these histopathological alterations remain unclear. Future research should incorporate longitudinal studies with extended follow‐up periods to assess the progression of tissue damage over time. In addition, advanced molecular techniques such as proteomic profiling of target tissues may elucidate the pathways involved and identify early biomarkers of toxicity. Applying these methodologies will strengthen mechanistic insights and enhance the translational relevance of findings, ultimately guiding safer therapeutic practices in avian veterinary medicine.

## Author Contributions

**Fatih Hatipoglu**: conceptualization, investigation, methodology, resources, project administration, supervision, writing – original draft, writing – review and editing. **Ayday Cunusova**: investigation, methodology. **Nariste Kadiraliyeva**: investigation, methodology. **Nur Abdimanap Uulu**: investigation, methodology. **Burak Mete**: investigation, methodology. **Orhan Corum**: writing – original draft, writing – review and editing. **Kamil Uney**: conceptualization, investigation, resources, writing – review and editing, writing – original draft, methodology, supervision.

## Ethics Statement

Approval was obtained from the Kyrgyz‐Turkish Manas University Faculty of Veterinary Medicine Ethics Committee (Date: 28/10/2021, No.: 2021/03).

## Consent

Written informed consent has been obtained from the animal owner to publish this paper.

## Conflicts of Interest

The authors declare no conflicts of interest.

## Data Availability

The original contributions presented in this study are included in the article. Further enquiries can be directed to the corresponding authors.
